# Chronic Post-stroke Psychosis with Left Cortical and Bilateral Inferior Cerebellar Involvement

**DOI:** 10.7759/cureus.6437

**Published:** 2019-12-21

**Authors:** Dominic Parfianowicz, Eduardo D Espiridion

**Affiliations:** 1 Pediatrics, Philadelphia College of Osteopathic Medicine, Philadelphia, USA; 2 Psychiatry, Reading Hospital-Tower Health, West Reading, USA

**Keywords:** neuropsychiatric symptoms, post-stroke psychosis

## Abstract

Post-stroke psychosis is the presence of delusions and/or hallucinations that result from an infarct in the cerebrovascular network. Involvement of a predominantly right-sided cortical pathology has been described in triggering the psychosis. In identified cases, patients often have little to no prior psychiatric history. We report a case of a 70-year-old female with chronic post-stroke psychosis consisting of auditory hallucinations and persecutory delusions. Our patient serves as a unique case in not only contributing to the limited number of documentations overall, but also in highlighting a presentation with infarction of the left parietal-temporal-occipital cortex and bilateral inferior cerebellum.

## Introduction

Psychosis, the presence of delusions and/or hallucinations without insight, can be extremely debilitating to life [1]. It is described as primary when associated with a psychiatric disorder such as any schizophrenia spectrum disorders or mood disorders. It is instead designated to be secondary if caused by another medical condition. Perhaps one of the more interesting types of secondary psychosis is post-stroke psychosis. Post-stroke neuropsychiatric symptoms in general often go undiagnosed but are thought to affect around 30% of cerebrovascular accident (CVA) survivors [2]. Prevalence of psychosis specifically is relatively rare in the post-stroke setting, having been estimated to occur in approximately 4% of patients [2,3]. The affected brain region seen on imaging in conjunction with the associated psychotic symptoms can help to advance the understanding of how and where in the brain an insult to specific connections relates to the pathogenesis of the induced psychosis. A collection of case reports has shown delusions and hallucinations primarily arising after a right hemisphere lesion with involvement of the frontal, temporal, and parietal areas being most common [2-4].

Here we report a case of chronic post-stroke psychosis in a 70-year-old female who presented to the hospital for monitoring of acute chest and left upper extremity pain and associated dyspnea. She reported a five-year history of auditory hallucinations and persecutory delusions following a CVA. Brain scanning with computerized tomography (CT) and magnetic resonance imaging (MRI) showed the presence of an old infarct in the left parietal-temporal-occipital lobe along with minor infarcts in the inferior cerebellar hemispheres bilaterally.

## Case presentation

This is a case of a 70-year-old Caucasian female with no personal psychiatric history prior to a CVA but a positive family history for mostly unspecified psychiatric disorders in her immediate relatives and also her son who had “serious substance-abuse issues” and was recently diagnosed with schizophrenia. Her medical history included essential hypertension, hyperlipidemia, type 2 diabetes mellitus, paroxysmal atrial fibrillation, coronary artery disease status post-coronary artery bypass graft, and stroke.

She was admitted to the hospital for observation of chest and left upper extremity pain and dyspnea with telemetry monitoring, serial troponins, and a cardiology consultation. Psychiatric consultation was requested after the patient reported hearing voices. Upon interview, she stated that she had been suffering from auditory hallucinations and paranoid delusions for the past five years, which she said began after a CVA. She initially did not want to seek help in fear of being labeled as “crazy.” She described the auditory hallucinations as constant indistinguishable voices that were making her anxious and confused and were negatively affecting her concentration and quality of sleep. Her main complaint was the increasingly bothersome insomnia. The patient also expressed concerns that her food was being poisoned and that someone else besides the nursing staff was following her. She denied any drug use, admitting to only an occasional use of alcohol but none recently. She reported no prior sexual or physical abuse. She did not have any feelings of depression or suicidal ideation and exhibited no episodes of aggression. She was not cognitively impaired and denied any symptoms suggestive of mania or hypomania. The patient also mentioned experiencing continual residual effects of numbness and discomfort in the right upper and lower extremity from the associated CVA.

**Figure 1:**
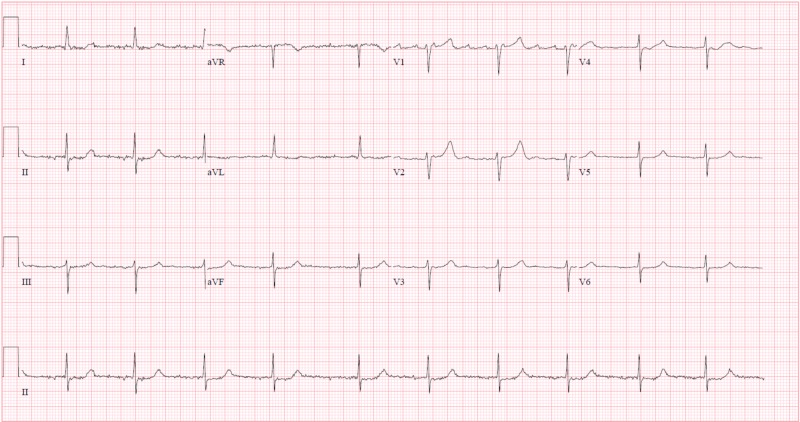
Electrocardiogram An electrocardiogram done during this hospitalization was interpreted as rate-controlled atrial fibrillation.

**Figure 2:**
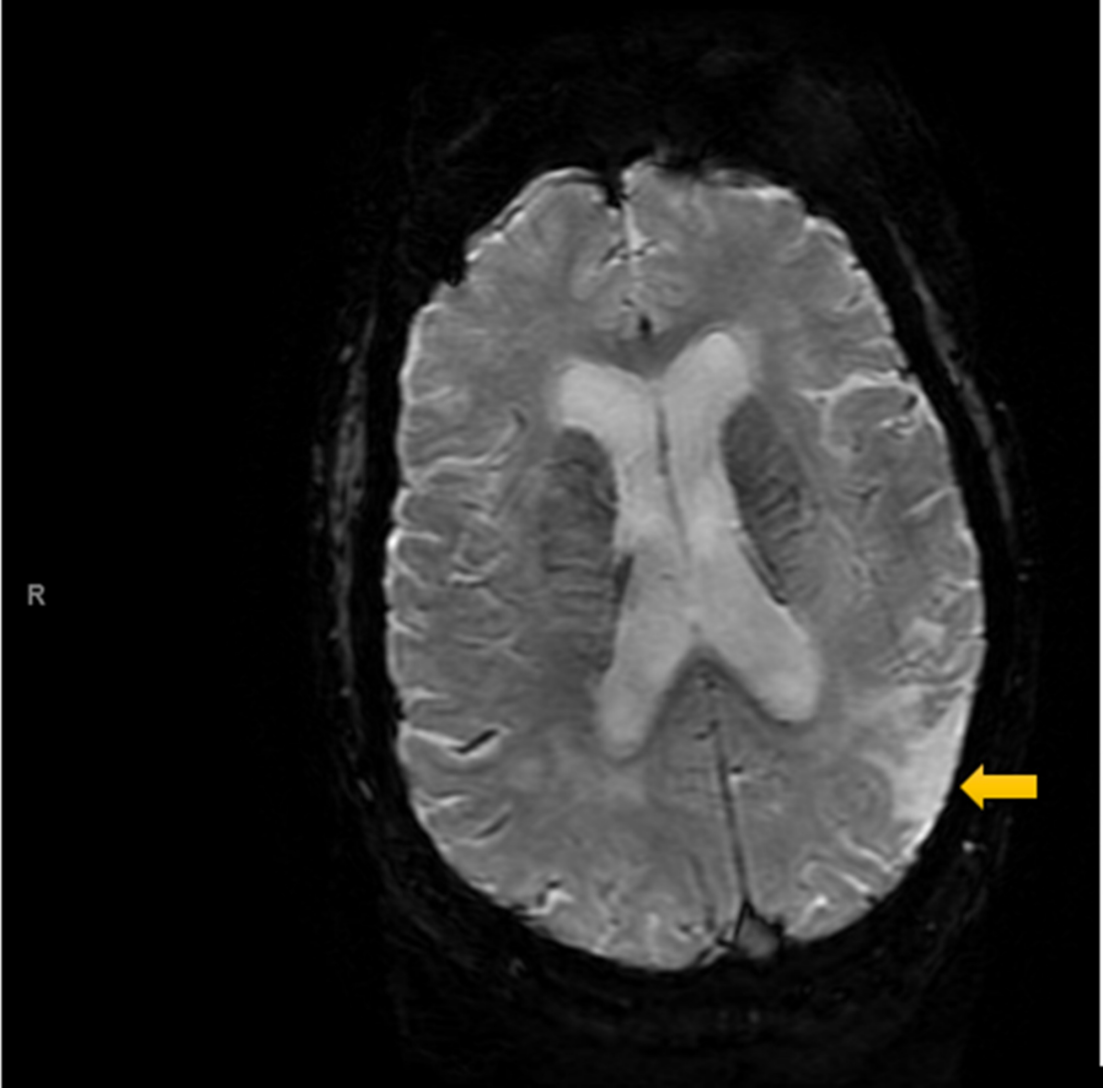
Susceptibility-weighted MRI Scan of the Head Non-contrast susceptibility-weighted MRI showing an old infarct in the left parietal-temporal-occipital lobe.

**Figure 3:**
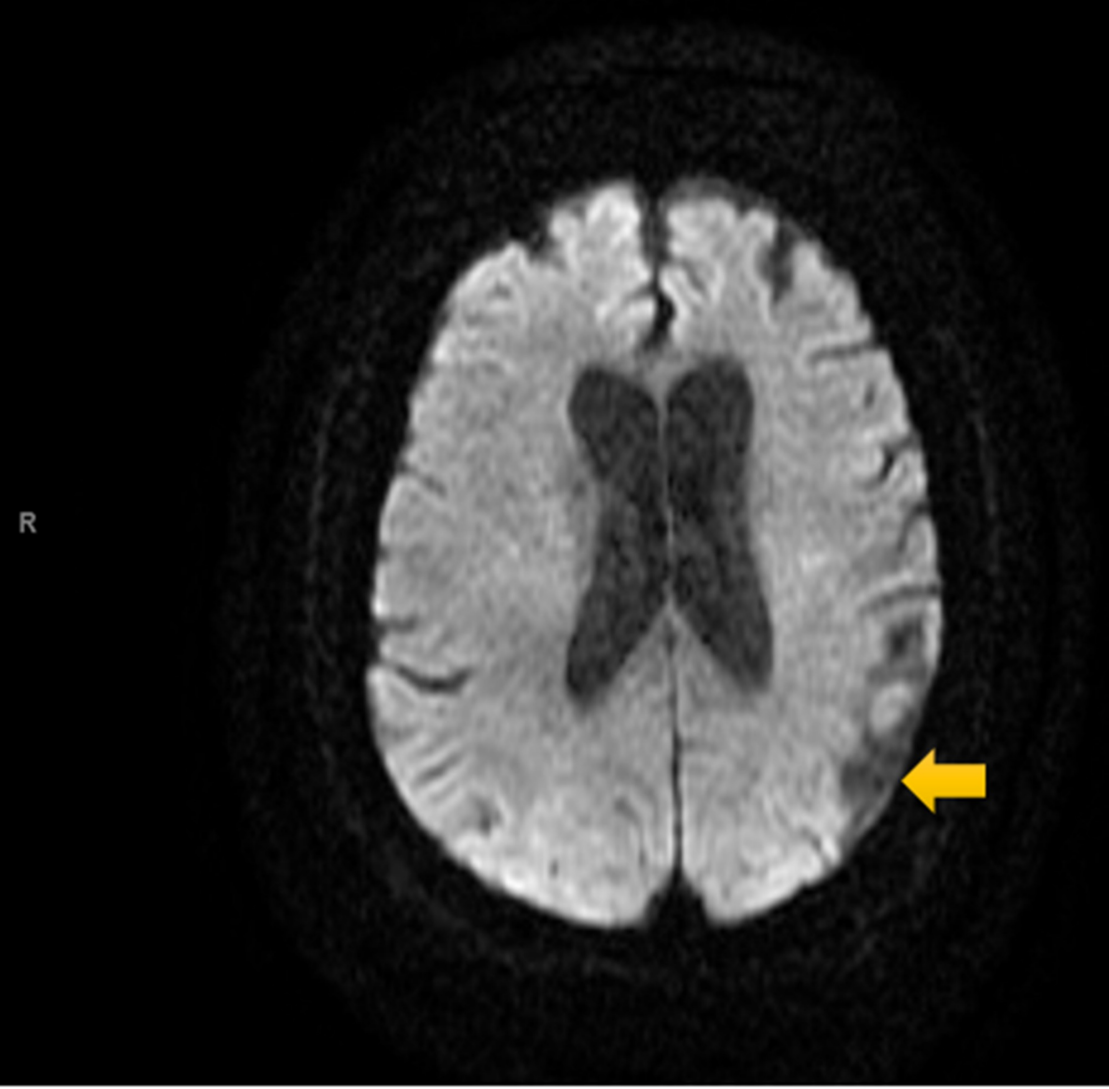
Diffusion-weighted MRI Scan of the Head Non-contrast diffusion-weighted MRI showing an old infarct in left parietal-temporal-occipital lobe.

**Figure 4:**
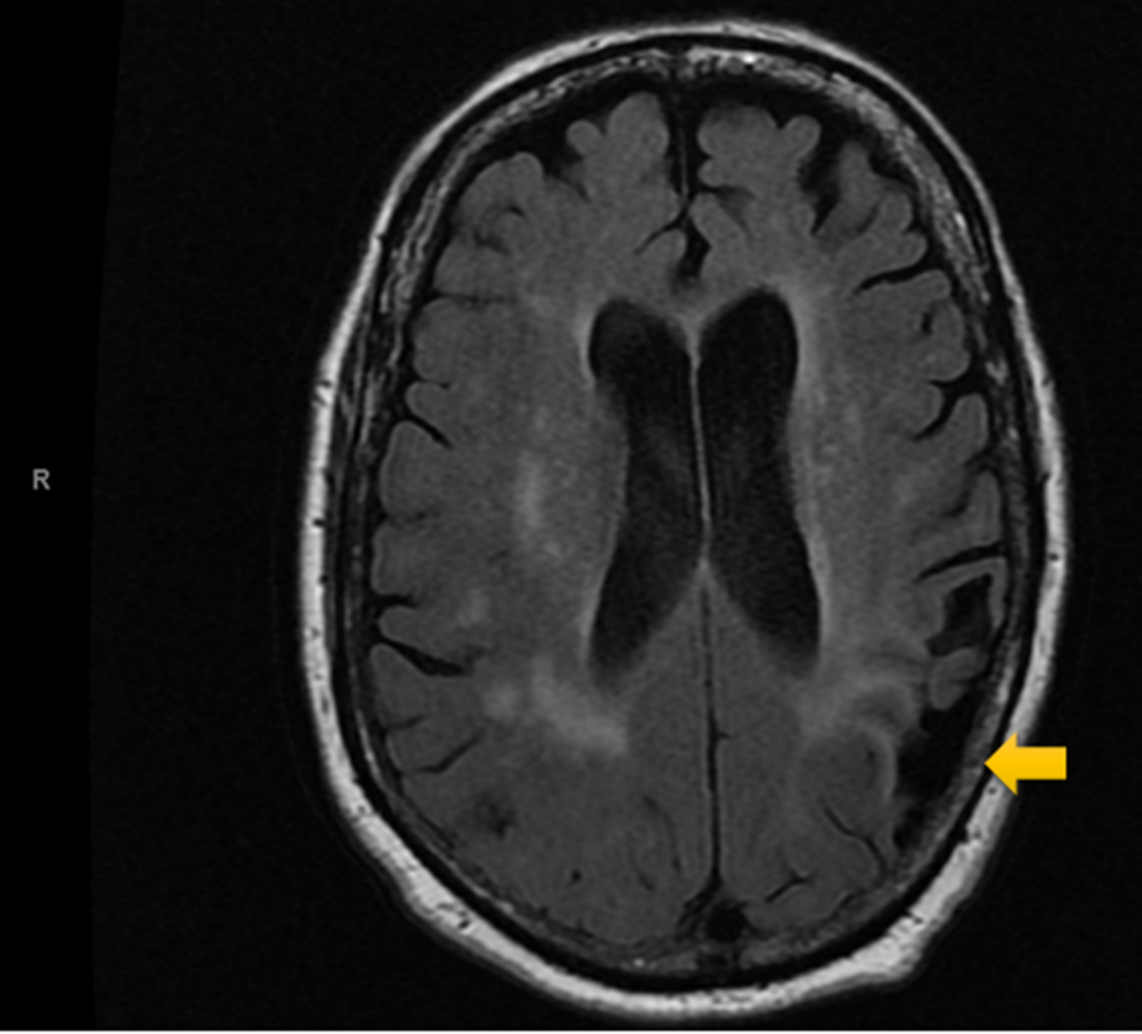
T2-flair MRI Scan of the Head Non-contrast T2-flair MRI showing diffuse cerebral atrophy along with point atrophy in the area of the suspected CVA in the parietal-temporal-occipital lobe. CVA, cerebrovascular accident.

**Figure 5:**
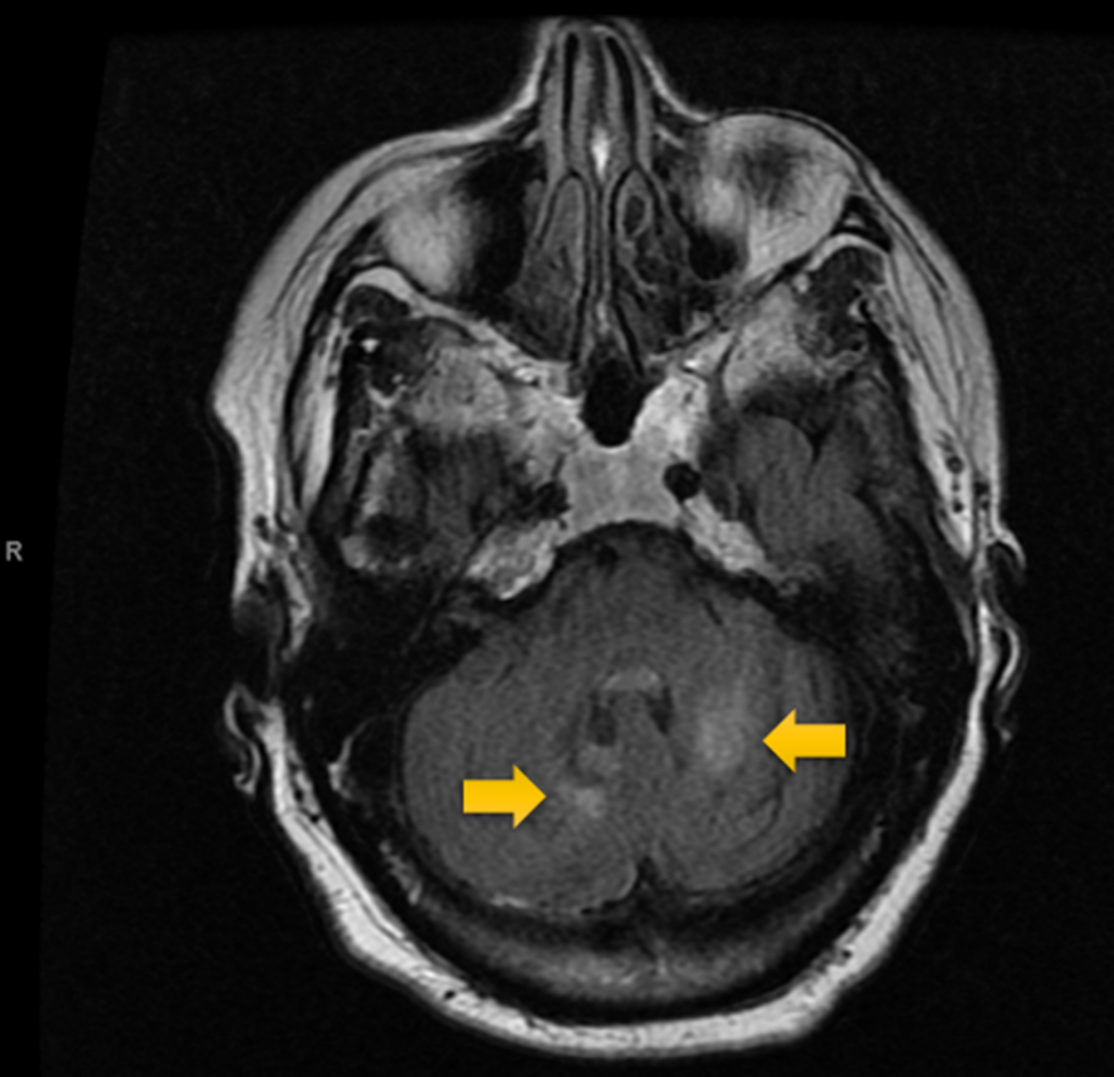
T2-flair MRI Scan of the Head Non-contrast T2-flair MRI showing old infarcts bilaterally in the inferior cerebellar hemispheres.

**Figure 6:**
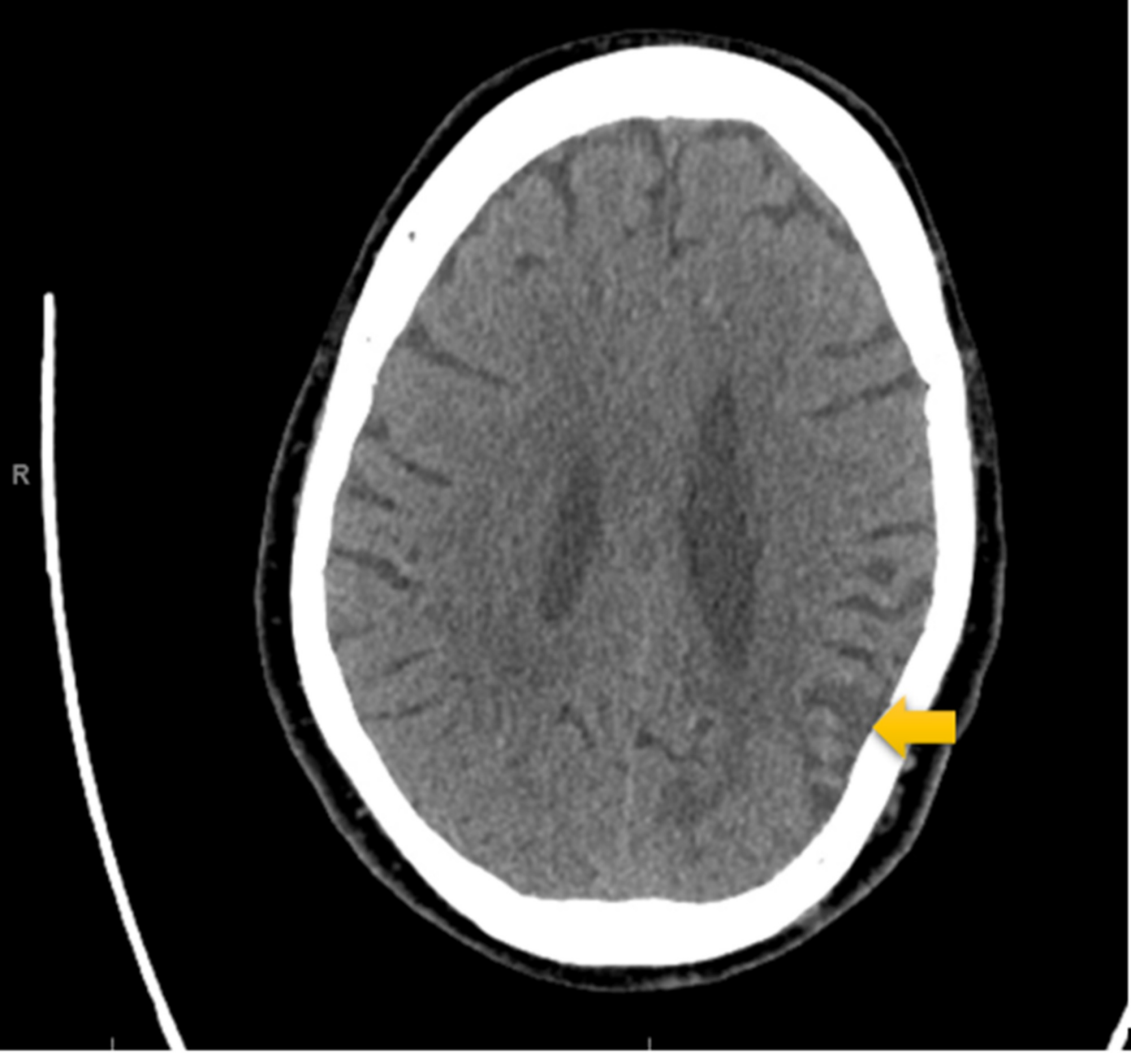
Non-contrast CT Scan of Head A non-contrast CT scan of the cerebral cortex from a few months prior to this hospitalization. Remote areas of infarction were described in the left parieto-occipital lobe.

Electrocardiography (ECG, Figure 1) was interpreted as rate-controlled atrial fibrillation. Serial troponins were negative throughout. All labs were within normal limits except for glucose and magnesium. The patient’s glucose was recorded at 254 mg/dL, while magnesium level was mildly decreased at 1.7 mg/dL at time of admission. A non-contrast MRI of the brain (Figures 2-5) done during this hospital stay for a focal neural deficit with a history of CVA suggested the presence of an old infarct in the left parietal-temporal-occipital lobe and smaller infarcts in the inferior cerebellar hemispheres bilaterally. A non-contrast CT scan of the head (Figure 6) from three months prior was interpreted similarly. No acute infarcts or acute pathologic processes pertaining to the brain were noted on these imaging modalities. The left upper extremity pain our patient presented with during this hospital stay was concluded to be likely secondary to a radicular neuropathy.

The patient was diagnosed with an unspecified residual psychosis disorder due to stroke and was subsequently started on quetiapine 12.5 mg nightly. Upon discharge from the hospital after reconciliation of the chest pain and dyspnea two days later, she was referred for follow-up with outpatient cardiology and psychiatry.

## Discussion

Psychiatric symptoms in the post-stroke time period are not uncommon with depression being the most frequent occurrence [2,5]. Post-stroke psychosis, however, is a relatively rare phenomenon that has therefore mostly been reported via a finite number of case reports. Imaging analysis can be extremely useful in delineating the relationship between brain pathology and post-stroke psychosis. The most frequently reported type of psychosis following a stroke was delusional disorder to which a predominance of case reports attributed a right hemisphere lesion of the frontal, temporal, and/or parietal lobes [2-4]. Visual hallucinations were found to be more associated with occipital strokes, while auditory hallucinations were thought to be more common with a subcortical stroke, but have also occurred with strokes affecting the cortex [5]. Interestingly, a dysfunction of the cerebellum has been increasingly implicated in first-episode psychosis, including that related to schizophrenia [6]. In fact, a significant reduction in cerebellar volume has been observed in patients with schizophrenia [6]. It is therefore not surprising that infarction of areas such as the cerebellum, thalamus, basal ganglia, and midbrain has also been described in the context of post-stroke psychosis [2,7,8].

Our patient with extensive medical history expressed a five-year duration of psychotic symptoms after a CVA. With the associated stigma of neuropsychiatric disorders of any form, patients may elect to not seek professional help. Hence, it is fairly common that these symptoms are underdiagnosed and subsequently untreated [5,9].

Imaging analysis can help to correlate our patient’s reported symptoms of auditory hallucinations and persecutory delusions with the stroke-affected brain areas. A non-contrast MRI and non-contrast CT (Figures 2-5, Figure 6, respectively) displayed the presence of past infarcts to the bilateral inferior cerebellar hemispheres and the left parietal-temporal-occipital lobe. Insult to a functionally connected brain network made up by neural routes between the cerebellar vermis, inferior cerebellum, and right superior temporal sulcus has been described in the pathogenesis of hallucinations [8,10,11]. It is therefore reasonable to conclude that an infarct affecting any of these connections can play a role in the emergence of hallucinations following a stroke. Auditory hallucinations, in particular, were also linked to lesions of the cerebellar dentate nucleus [10]. The presence of a lesion in the left parietal-temporal-occipital lobe as seen in our patient is unique and not well described in the literature. The lesion appears to border, if not include, part of the left Heschl gyrus, also known as the transverse temporal gyrus. It is one of the first cortical structures involved in perception of auditory information. Some studies have noted a smaller volume of the left Heschl gyrus in patients with paranoid schizophrenia [12]. In addition, patients with delusions in the post-stroke time period were found to most consistently present with a posterior temporoparietal infarct [2,3]. This may help to clarify some of the persecutory delusions experienced by our patient.

In regard to our patient and her family psychiatric history, the diagnosis of schizophrenia in her son suggests a potential genetic susceptibility for psychiatric abnormalities in our patient even with an unremarkable prior personal psychiatric history. The occurrence of the stroke in our patient may have further advanced this possible genetic predisposition by affecting the neural connections which manifested as hallucinations and delusions following the incident.

There have been several risk factors implicated in post-stroke psychosis, many of which are concurrent risk factors for stroke. Of these, hypertension, hyperlipidemia, and diabetes mellitus were the most commonly reported [2,8]. Our patient had each of these risk factors and, with a measured glucose level of 254 mg/dL, likely did not have appropriate control of those conditions whether that be from inadequate medication or non-adherence. Interestingly, another risk factor our patient held was a prior cardiac procedure [2].

Treatment of post-stroke psychosis involves use of antipsychotic medication. The psychosis may be temporary in some patients while remaining persistent in others. In general, it can be treated in a similar fashion to primary psychosis [3,13]. In our described case, quetiapine 12.5 mg nightly was prescribed to address both the psychosis and the patient's reported insomnia with the plan to further treat her post-stroke psychosis in the outpatient psychiatry setting if she indeed wanted to seek therapy. Quetiapine, along with most other antipsychotics, comes with a warning of QTc interval prolongation and possible subsequent torsade de pointes. Risk of QTc prolongation with the use of a recommended therapeutic dose of quetiapine has been reported to be minimal among patients without other risk factors such as a compromised metabolism, electrolyte abnormalities, congenital prolonged QTc, or co-administration of QTc-prolonging drugs [14]. Our patient, although having an extensive medical history, was not diagnosed with any acute disease processes as she was admitted for observation. Because the ECG was stable with a normal QTc interval along with most of the lab chemistry values being within normal limits, a minimal dose of quetiapine was regarded as appropriate for initiating treatment.

## Conclusions

The patient history of psychotic symptoms after a CVA in correlation with imaging interpretation suggested a diagnosis of chronic post-stroke psychosis. There is increasing documentation for the role of an infarcted inferior cerebellum in the pathogenesis. Our patient's unique case further contributes to this mounting evidence and also presents a post-stroke psychosis scenario with left cortical involvement which is uncommon.
